# Simulating cardiac signals on 3D human models for photoplethysmography development

**DOI:** 10.3389/frobt.2023.1266535

**Published:** 2024-01-10

**Authors:** Danyi Wang, Javaan Chahl

**Affiliations:** ^1^ UniSA STEM, University of South Australia, Mawson Lakes, SA, Australia; ^2^ Platforms Division, Defence Science and Technology Group, Edinburgh, SA, Australia

**Keywords:** simulated cardiac signal, imaging photoplethysmography (IPPG), bionic human model, non-contact, synthetics

## Abstract

**Introduction:** Image-based heart rate estimation technology offers a contactless approach to healthcare monitoring that could improve the lives of millions of people. In order to comprehensively test or optimize image-based heart rate extraction methods, the dataset should contain a large number of factors such as body motion, lighting conditions, and physiological states. However, collecting high-quality datasets with complete parameters is a huge challenge.

**Methods:** In this paper, we introduce a bionic human model based on a three-dimensional (3D) representation of the human body. By integrating synthetic cardiac signal and body involuntary motion into the 3D model, five well-known traditional and four deep learning iPPG (imaging photoplethysmography) extraction methods are used to test the rendered videos.

**Results:** To compare with different situations in the real world, four common scenarios (stillness, expression/talking, light source changes, and physical activity) are created on each 3D human. The 3D human can be built with any appearance and different skin tones. A high degree of agreement is achieved between the signals extracted from videos with the synthetic human and videos with a real human-the performance advantages and disadvantages of the selected iPPG methods are consistent for both real and 3D humans.

**Discussion:** This technology has the capability to generate synthetic humans within various scenarios, utilizing precisely controlled parameters and disturbances. Furthermore, it holds considerable potential for testing and optimizing image-based vital signs methods in challenging situations where real people with reliable ground truth measurements are difficult to obtain, such as in drone rescue.

## 1 Introduction

Detecting cardiac signals based on image sequences provide a non-contact means for healthcare monitoring that can improve the lives of millions of people. Existing image based vital signs estimation methods can be divided into two main categories: imaging photoplethysmography (iPPG) and imaging ballistocardiography (iBCG). iPPG is a set of techniques that aim to recover the changes of volume and oxygen saturation in blood close to the surface of the skin, the resulting signal is known as the blood volume pulse (BVP). While iBCG-based techniques aim to extract subtle motion of the body caused by the mechanical flow of blood and the respiratory system.

In 2008 (Verkruysse et al., 2008), pioneered the extraction of PPG signals from a human face using a standard digital camera. Subsequently, in 2013, iBCG signals were successfully extracted from head motion (Balakrishnan et al., 2013). Since then, numerous algorithms based on the techniques above have been proposed to enable recovering cardiac signals from image sequences. In general, they can be summarized as traditional algorithms and deep learning algorithms. Traditional algorithms use modeling methods based on optical principles [such as POS (Wang et al., 2016)] or signal processing methods [like ICA (Poh et al., 2010) or PCA (Lewandowska et al., 2011)] to convert useful spatial and temporal information in ROIs from videos to extract cardiac signals. These cardiac signals are all extracted from subtle light changes which are easily masked by body motion and lighting fluctuations. Given that deep learning techniques can adequately model the dynamic spatial and temporal information present in videos, they have shown impressive performance over traditional source separation algorithms [e.g., DeepPhys (Chen and McDuff, 2018); PhysNet (Yu et al., 2019); TS-CAN (Liu et al., 2020); EfficientPhys (Comas et al., 2022)]. It is well known that reliable neural network models are highly dependent on extensive and representative data sets for training. However, variables like motion, changes in illumination, and variations in skin type collectively introduce intricate influences on image based cardiac signal extraction.

Collecting a high-quality data set with accurate physiological parameters for models to learn from is a big challenge. The datasets for image-based cardiac signal measurements are privacy-sensitive, since most video recordings include the participant’s face and sensitive physiological signals, which increases the difficulty of recruiting and organizing participants. Moreover, deep learning models are hungry for the availability and quality of training datasets. Thus, how to accurately control the variables such as motion, lighting changes, and different skin types during data recording processes is also a huge challenge. So far, the public data sets intended for image based heart rate estimation are either limited in size or not diverse. To overcome these limitations, Niu et al. (2018) introduced a heart rate estimator that was pre-trained on synthetic spatio-temporal maps for cardiac signals. Similarly, Song et al. (2020) present a convolutional neural network (CNN) model trained on spatio-temporal heart rate (HR) feature images. These HR feature images were built from synthetic pulse signals that were generated based on real ECG signals. However, data sets based on 2D data sources do not contain all of the phenomena found in the real world. In 2022, McDuff et al. (2022a) first implemented facial blood flow changes into synthetic avatars and generated samples under a range of real-life conditions. Results show that models trained on synthetic and real video data improve the quality of recovered cardiac signals. Based on this technology, a synthetic dataset of 2,800 videos called SCAMPS was launched in the same year (McDuff et al., 2022b).

Additionally, several remote PPG toolboxes [pyVHR (Boccignone et al., 2022), rPPG (Liu et al., 2022) and PhysBench (Wang et al., 2023)] have been deployed to replicate and test deep learning and traditional methods with supporting public benchmark datasets. In particular, rPPG and PhysBench have trained and validated the SCAMPS dataset on different deep learning algorithms. Although the SCAMPS dataset has a large amount of simulation data, compared with other real training sets, the benchmark results of models trained on it do not show outstanding accuracy and robustness, particularly in some cases such as the MMPD dataset (Tang et al., 2023) which contains darker skin type videos. One reason might be that although the SCAMPS dataset was much more diverse than real training sets, the size of each variable group is relatively smaller. So it is possible to cause larger errors on some specific subjects (e.g., darker skin or larger motion) when the model reaches a local minima (McDuff et al., 2022a). Another reason might be that there is still a gap between simulation and real videos (McDuff et al., 2022b), such as the avatars in SCAMPS only have PPG signals, but BCG signals have also been proven to affect the accuracy of iPPG signal recovery.

In this study, we propose an enhanced 3D Human Model which has a cardiac signal with similar dynamics to a real person to solve the challenge of building controlled yet diverse data sets for machine learning. The model can be used to test image-based vital signs methods under any environmental condition that can be modelled and rendered, including environment, light and movement, all repeatable and controllable. The main contributions of this study are: 1) the 3D Human Model has a complete body, and all subject variables (such as expressions, blinks, skin types, physical activities, etc.) and environmental variables (light changes) can be systematically controlled in the simulation environment; 2) we integrated body movements caused by involuntary movements and breathing into the 3D human body, making the 3D model objectively more similar to real people; 3) evaluate the performance on a set of bionic humans with different appearances against real database videos, by using traditional methods and machine learning methods, specifically testing some special variables such as body movement and people with darker skin types.

The remainder of the paper is as follows. In Section 3, we present the imaging system math model for human skin and how to simulate physiological signals, then integrate them into the 3D model. In Section 4 the proposed model is experimentally evaluated and compared. Then we discuss possibilities, extensions and limitations in Section 5. Conclusions and future work are in Section 6.

## 2 Related work

### 2.1 Public datasets of real humans

The first public database used for remote vital signs estimation was MAHNOB-HCI (Soleymani et al., 2011), which contains 527 videos of 30 subjects with their reference data, recorded with small facial movements under controlled illumination. Similarly (Zhang et al., 2016), introduced the MMSE-HR database, which consisted of 560 videos of 140 subjects with synchronous heart rate reference data involving facial expression changes. However, these two databases were all originally designed for emotion analysis as with DEAP (Koelstra et al., 2011) which consists of 160 videos captured from 32 participants synchronized with physiological parameters such as blood pressure and breathing rate. There are also some public-databases especially designed for the task of remote vital signs estimation. In 2014, Stricker et al. (2014) released the PURE database consisting of 60 videos from 10 subjects, in which all of the subjects were asked to perform six kinds of movements such as talking or head rotation. Another publicly available dataset UBFC-RPPG (Bobbia et al., 2019) includes 43 videos synchronized with a pulse oximeter finger clip sensor. Subjects sat stationary in front of the camera while at same time they were required to play a time sensitive mathematical game that supposedly raises the heart rate. Li et al. (2018) proposed the OBF database which was specifically designed for heart rate variability (HRV) feature analysis, the data were recorded both from healthy subjects and from patients with atrial fibrillation (AF). However access to this database is not free. To complement the limited diversity of large movements in public datasets, a dataset called ECG-Fitness (Špetlík et al., 2018) was collected with the subjects performing on fitness machine. In 2023, MMPD dataset (Tang et al., 2023) has been published to broaden the diversity of facial appearances and lighting conditions. In particular, MMPD dataset is the first public dataset that includes subjects with diverse skin types (Fitzpatrick scale of 3–6).

Table 1 summarizes some properties of recent public datasets. While more and more real human datasets for remote vital signs detection have been developed in recent years, none of them currently contain sufficient features to enable the deep learning model reliably achieve generalizability. Some of the datasets also exhibit data synchronization issues (Yu et al., 2019; Comas et al., 2022; Wang et al., 2023). The synchronization between videos and sensor signals significantly impacts training performance.

**TABLE 1:** A summary of real human datasets.

Real human database	Subjects	Videos	Illumination	Motion	Gold standard
DEAP Koelstra et al. (2011)	32	180	Lab Environment	Expression	EEG/PPG/BP/BR
MAHNOB-HCI Soleymani et al. (2011)	27	527	Lab Environment	Expression	EEG/PPG/BP/BR
PURE Stricker et al. (2014)	10	60	Lab Environment	Talking	PPG
AFRL Estepp et al. (2014)	25	300	Lab Environment	Head POS	PPG
COHFACE Heusch et al. (2017)	40	160	Lab + Nature	Stable	PPG
OBF Li et al. (2018)	106	2120	Lab Environment	Stable	PPG/ECG/BR
ECG-Fitness Špetlík et al. (2018)	17	204	Lab Environment	Physical Activities	ECG
UBFC-RPPG Bobbia et al. (2019)	42	42	Lab Environment	Stable	PPG
VIPL Niu et al. (2019)	107	2378	Lab Environment	Head POS	PPG
MMPD Tang et al. (2023)	33	660	Lab + Nature	Head POS/Expression + Talking	PPG/BR
RLAP Wang et al. (2023)	58	754	Lab Environment	Head POS/Expression + Talking	PPG

### 2.2 Synthetic iPPG video

The technology for creating synthetic iPPG videos can avoid data synchronization issues and does not require a lot of human and material resources to complete. Currently, there is a few of ongoing research focused on the generation of synthetic videos. In 2020, Tsou et al. (2020) made a first attempt to create synthetic videos by merging rPPG signals into given source images/videos using augmentation methods. Ba et al. (2022) introduced another augmentation method in which they employed a generative neural network to transform real patient skin tones to a variety of skin tones. Both methods fall into the category of “semi-synthetic” methods (McDuff et al., 2023) and heavily rely on real human samples. The first synthetic dataset, SCAMPS (McDuff et al., 2022b), was published in 2022. It generated synthetic iPPG video by using graphics-based technology, leveraging human physical models to create a diverse and realistic representation. The facial identities are created from a combination of 3D face scans from publicly available sources (3dscanstore) and physically-based shading material in Blender. The input PPG signal for the avatars is generated through the convolution of a Gaussian window with the beat sequence, derived from a heart rate frequency range. In contrast to the intricate pipeline of SCAMPS, Wang et al. (2022) presents a more user-friendly approach for generating synthetic videos. This method employs a statistical 3D head model, extracting facial features from publicly available in-the-wild face datasets [BUPT-Balancedface (Wang et al., 2019)], while the PPG waveforms are recorded from real human subjects.

Table 2 summarizes some properties of recent public synthetic datasets and our bionic human set.

**TABLE 2:** A summary of synthetic databases.

Sythestic database	Model type	Subjects	Videos	Illumination	Motion
SCAMPS McDuff et al. (2022b)	face rig	2800	2800	Lab + Nature	Expression/Talking/Head POS
Wang et al. (2022)	face rig	480	480	Lab	Head POS
our bionic human dataset	face rig + body rig	12	34	Lab + Nature	Expression/Talking/Physical Activities

## 3 Materials and methods

To simulate realistic cardiac signal in a 3D Human Model, there are four major steps: 1) imaging system model; 2) generating a synthetic cardiac signal; 3) generating the body movement signal; 4) integrating the synthetic cardiac signal into the human skin model.

### 3.1 Imaging system model

Normally, a simple imaging model can be expressed as Eq. 1:

$$f\left(x,y\right)=i\left(x,y\right)r\left(x,y\right)$$ (1)

where *i* is the incident light component and *r* is the reflected component; *x*, *y* are the coordinates in the image. For human skin, *r*(*x*, *y*) includes specular (mirror-like) light reflection from the skin surface reflection and diffuse reflection.

**FIGURE 1:**
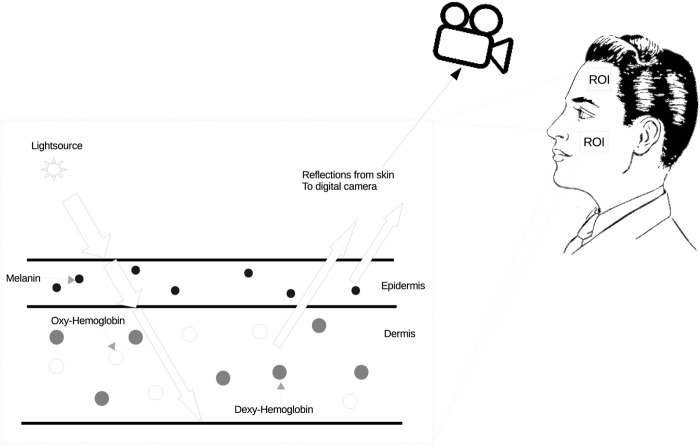
Method for skin color changes. The spectrum of hemoglobin absorption in blood cells is different between the oxygenated state and the deoxygenated state, and oxygenated blood is brighter than deoxygenated blood. In addition, the color of oxygenated blood is lighter than that of deoxygenated blood.

As illustrated in Figure 1, the alteration in blood color, influenced by the exchange of gases in the heart and lungs, directly affects the skin color. These subtle changes can be captured by a standard digital camera. The iPPG signal is derived from these subtle skin color variations. Consequently, by integrating the aforementioned simple image model, the cardiac signal model over time via a sequence of frames can be defined as Eq. 2:

$$C=I\cdot r\left(t\right)+N\left(t\right)=I\cdot \left(p\left(t\right)+m\left(t\right)\right)+N\left(t\right)$$ (2)

where *I* denotes the lighting variations; *p*(*t*) is diffuse reflection variation which has the pulsatile information, the iPPG signals, from the RGB channel respectively; *m*(*t*) is skin specular reflection variation which is caused by body motion; *m*(*t*) cannot be ignored, because even when stationary, the human body still has slight movement due to the body’s balance control. From the bio-mechanical standpoint, a simple body movement system can be described as a sequence of stacked inverted pendulum motions. We explained this part more in 3.3. *N*(*t*) is white noise mainly caused by the camera.

### 3.2 Synthetic iPPG generated

**FIGURE 2:**
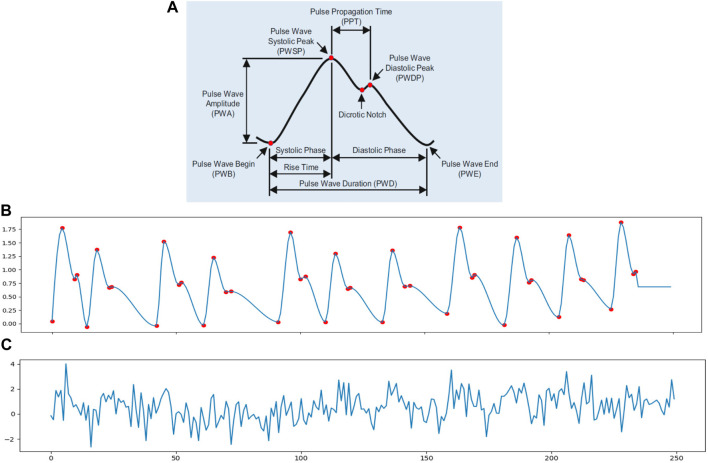
**(A)** Pulse waveform of photoplethysmogram (PPG) (Fischer et al., 2017). **(B)** A 10 s synthetic ppg signal with heart rate of 70 beats/min and 0.3 Hz inter-beat interval (IBI) variation; the sample rate is 25 Hz. **(C)** A simulated iPPG signal based on **(B)**.

In order to simulate the cardiac signal in video, we need to first generate the iPPG signal. A standard PPG wave is shown in Figure 2A. The analysis of the PPG signal has been used to measure the vital signs like heart rate, respiration rate, heart rate variability (HRV), oxygen saturation, blood pressure and to detect some vascular diseases (Allen, 2007). There are many different methods to generate the PPG signal such as modelling the PPG waveform by Gaussian functions (Banerjee et al., 2015; Tang et al., 2020), generate the pulse signals via sinusoidal signals (Wannenburg and Malekian, 2015; Niu et al., 2018) and a synthetic PPG signal based on ECG signals (Sološenko et al., 2017; Song et al., 2020). However, these PPG modeling approaches were too complex for this study because they included more physiological assumptions than we needed. Also, simple equations only using sinusoidal signals contain too little information to even express HRV.

Thus, in order to meet the need to control some basic parameters in the cardiac signal, in this study, we chose to use an open source python library neurokit2 (neurokit2) to generate the simulated PPG signal (*ppg simulate* in neurokit2). The *ppg simulate* uses a four-point interpolation method to simulate the PPG signal. From Figure 2 we know that a PPG wave can be described with four key-points: wave onset, location of the systolic peak, location of the dicrotic notch and location of the diastolic peak. The PPG signal is then generated by interpolating the key-points with a cubic curve at the desired sampling rate. It can control the parameters like HRV, PR, peak position and motion artifacts to make the PPG signal more realistic. Figure 2B shows a simulated PPG signal *p*(*t*) via function *ppg simulate* neurokit2 with basic physiological information: heart rate, breathing rate and HRV. The red dots present the key-points for interpolation nodes to generate the synthetic signal. Figure 2C is an example of iPPG signal with white noise *N*(*t*).

### 3.3 Generating the involuntary body movement signal

To model *m*(*t*), we have classified involuntary movements of the human body into two main categories: BCG motion and movements of body parts associated with breathing.

#### 3.3.1 BCG motion

When people are stationery, whether standing or sitting, a swaying movement occurs in many parts of human body (such as neck, hip, and ankle); the frequency components of the body movement distribute in the area of lower 0.5 Hz (hip and ankle rotations) and beyond 0.9 Hz (anti-phase coordination), respectively (Kim et al., 2005; Morasso et al., 2019). Since BCG motion was found to be a source of artifacts in iPPG signals (Moco et al., 2015), in this model, we only consider the high frequency component that can be used for BCG signal analysis from video (Balakrishnan et al., 2013). Also, from Moco et al. (2015), the flexion and extension movements of the neck appears to have the strongest affect over iPPG signal. For our purposes, with a simplified motion function, the BCG motion signal is written as Eq. 3:

$$bcg\left(t\right)=\theta_{X}\left(t\right);$$ (3)

where *θ* is the rotation angle of the *X*-axis of the neck (0.12° ± 0.03°).

#### 3.3.2 Breathing

Breathing induces movements in the head, shoulders, and chest. In our 3D human simulation, we replicate these motions by manipulating the y-location of the head and shoulder rig (up and down) and the x-location of the chest rig (forward and backward). The breathing frequency is dynamically sampled within the range of 0.13–0.4 Hz (equivalent to 8–24 breaths per minute).

### 3.4 Integrating synthetic cardiac signal

**FIGURE 3:**
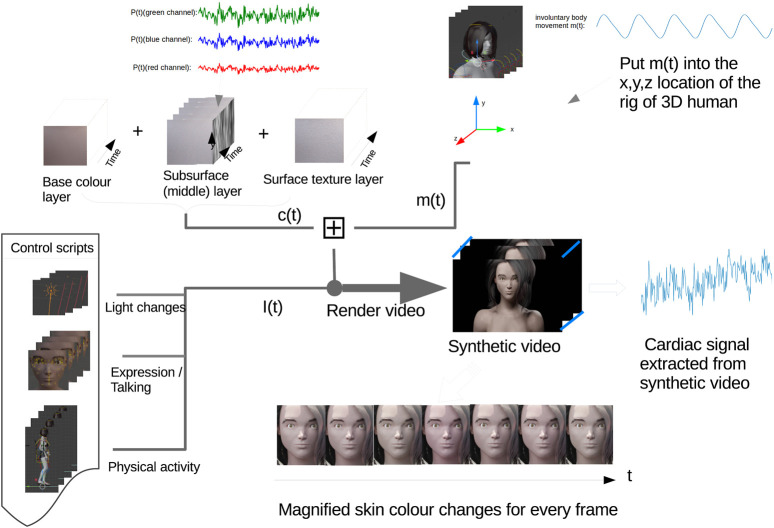
Framework of the integration method.

The 3D model was built in Blender (Blender). To realistically synthesize skin in Blender based on the anatomical structure and physiological function of real skin (Tsumura et al., 2003; Doi and Tominaga, 2006), the synthetic human skin was designed to consist of three layers: Base color layer, subsurface layer and surface texture layer. In the human skin model (see Figure 3), the base color layer is for melanin in the epidermis which controls the skin color of the avatar. The subsurface layer is a variable area which is designed to present the hemoglobin status change in the dermis as explained in Figure 1. To make the model more lifelike, we also added a surface texture layer to simulate the texture and wrinkle of realistic human skin. The facial and bodily rig has been built based on a human skeleton to control both facial actions (expressions, talking, and blinking) and body movements (iBCG motion, breathing, and physical activity).

To integrate a synthetic cardiac signal into the skin with every video frame, we first generate a synthetic PPG signal *p*(*t*) by using Toolbox neurokit2. Then the *p*(*t*) needs to normalize into the range of 0–1, which presents the rate of change of the cardiac signal in each of the red, green and blue channels in the subsurface layer. Considering spectral power distributions of visible light for the reflectance of the skin surface, we introduced *β*_*r*_, *β*_*g*_ and *β*_*b*_ as the weights for cardiac signals in each R, G, and B channel; where *β*_*r*_, *β*_*g*_, *β*_*b*_ are equal to 0.33, 0.77, and 0.53 respectively (As we know that the green channel has the strongest information from the cardiac signal (Verkruysse et al., 2008), we need to search for the accuracy weights of each color channel. The weighting [*β*_*r*_, *β*_*g*_, *β*_*b*_] = [0.33, 0.77, and 0.53] is from Wang et al. (2016) and De Haan and Van Leest (2014) which is based on ideal laboratory lighting conditions and the RGB camera). So the change to color induced by the cardiac signal in the subsurface layer can be written as Eq. 4:

$$c\left(t\right)=\left(\beta_{r},\beta_{g},\beta_{b}\right)P\left(t\right)$$ (4)

where *P*(*t*) is the simulated iPPG signal. At the bottom of Figure 3 we see the simulation of a physiological signal on a 3D model. After amplifying the pixel value for every frame, the Avatar’s skin color changes over time and can be clearly observed.

The final cardiac signal model which is extracted from the 3D model video is (Eq. 5):

$$C=I\cdot \left(c\left(t\right)+m\left(t\right)\right)$$ (5)

where *I* could be a constant *I*_0_ when people keep still under ideal laboratory lighting conditions or *I*(*t*) when there are illumination intensity changes. Figure 3 illustrates the framework for integrating skin color variation *c*(*t*) and involuntary body motion variation *m*(*t*) with the fluctuating illumination intensity *I*(*t*) (caused by light changes, facial movements or physical activity) into the animation.

### 3.5 Motion variation coding

To ensure precise control over motion variation, we break down each motion action into a single pattern. Each action pattern is regulated by the positioning or rotation of the body and face rig. These crafted patterns are subsequently integrated into the frames where action needs to be dynamically generated. Periodic actions, such as blinking and running/walking patterns, are repeated with the same duration across video frames. Non-periodic actions like smiling, laughing, or talking patterns are randomly distributed in the video frames. Additionally, variations in light source intensity, transitioning from brightness to darkness, are also introduced randomly. The algorithm is as follows (Algorithm 1):

Algorithm 1 Action algorithm.

  *framenum* = *M*⊳ Total number of frames to render
  **procedure** Periodic actions
    step = N ⊳ The frequency of action. The faster the action, the smaller the N.
   **for** i in range int (framenum/step) **do**
     actionInsertFrameKey = i* step
     <do pattern>
    **end for**
  **end procedure**
   **procedure** Non-Periodic actions
   i= 0
   **while** i ≤ framenum **do**
    step = random(0,j) ⊳ The frequency of action.
    actionInsertFrameKey = i+step
    <do pattern>
   **end while**
  **end procedure**

## 4 Experiments

### 4.1 Experimental setup

**FIGURE 4:**
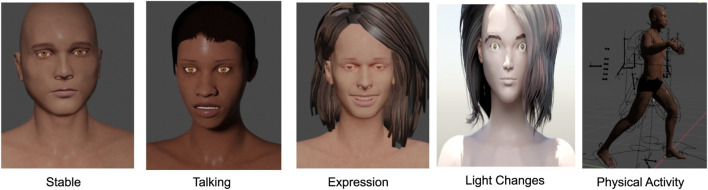
Examples of the appearances of the 3D human in different scenarios.

To compare with different situations in the real world, we rendered videos under four scenarios: 1) stationary person under stable laboratory light; 2) person with expressions/slight head movements/talking under stable laboratory light; 3) stationary person in a varying lighting environment; 4) person performing physical activities, such as walking and running (see examples in Figure 4). The stable laboratory light scenario corresponds to the constant *I*_0_ mentioned in Eq. 5 and induced changes in illumination, expression and physical activities correspond to *I*(*t*). The videos were rendered with 3D people with different heart and breathing rates. The involuntary body movement signal *m*(*t*) were set to the same frequency as the 3D human heart rate, which was the main source of artifacts in the simulated iPPG signal. We also introduced the HRV variations into the input cardiac signals for further study. All variables, including cardiac signals *c*(*t*), involuntary body movement signals *m*(*t*), and induced changes *I*(*t*), were imported into Blender through python scripts. The human mesh was generated by Blender free add-on MB-Lab. The video frame rates were rendered at 25 fps and the dimensions were 640*360 in every case.

#### 4.1.1 Image based iPPG methods

In this section, five traditional iPPG methods and four deep learning methods were used to process the 3D subject videos.

The traditional methods can be roughly classified into two groups: 1) signal processing methods; 2) skin pixel based methods. Signal Processing Methods: 1) **GREEN** (Verkruysse et al., 2008): the green channel has been shown to contain the strongest pulsatile/cardiac signal of the RGB channels (Verkruysse et al., 2008; Lee et al., 2013). The raw cardiac signal is the spatial average of the green color channel pixels in the facial area of a video; 2) **ICA** (Poh et al., 2010) is a Blind Source Separation (BSS) method which is based on ICA introduced by Poh et al. (2010). The ICA (JADE implementation) separates the raw signals into several independent signal sources. The second component of the signal sources is chosen to be the cardiac signal; 3) **CEEMDAN** (Al-Naji et al., 2017) use CEEMDAN to find out the iPPG signal from the raw signal averaged from the green channel pixels in the ROI of video sequences.

Skin Pixel Based Methods: 1) **CHROM** (De Haan and Jeanne, 2013): a linear combination of the chrominance signals from the bandpass filtered outputs of the spatial averaging in the ROI of the red, green and blue color channels respectively; 2) **POS** (Plan-Orthogonal-to-Skin) (Wang et al., 2016): defines a skin reflection model, which calculates a plane orthogonal to the skin-tone in the temporally normalized RGB space for iPPG extraction.

The deep learning methods we use to test the 3D model are **DeepPhys** (Chen and McDuff, 2018), **TS-CAN** (Liu et al., 2020), **PhysNet** (Yu et al., 2019) and **Physformer** (Yu et al., 2022). In which, DeepPhys and TS-CAN are two part 2D convolutional attention networks; PhysNet is a 3D convolutional network architecture; Physformer is an end-to-end video temporal difference transformer based architecture.

The code for the experiments was written in MATLAB and Python, some of the traditional methods source code refers to the iPhys Toolbox (McDuff and Blackford, 2019). The pretrained deep learning models are from rPPG (Liu et al., 2022), PhysBench (Wang et al., 2023) and Yu et al. (2022).

#### 4.1.2 Public datasets

Because there is no public dataset that contains all of the scenarios we need to test, we chose the public data set DEAP (Koelstra et al., 2011), ECG-Fitness (Špetlík et al., 2018) and MMPD dataset (Tang et al., 2023) as the control group. DEAP provides ground-truth scenes of stillness, expressions, and light source changes. ECG-Fitness is for physical activity validation and MMPD is used for testing darker skin tones. MMPD also offers stable and expression/talking scenarios for real people with darker skin types.

#### 4.1.3 Pre-trained models

Considering the data synchronization issues (Yu et al., 2019; Comas et al., 2022) of data set can affect the training performance. We use the RLAP (Wang et al., 2023) dataset which has been reported with no signal offset by Wang et al. (2023), as the training set for TS-CAN, DeepPhys and PhysNet. The pre-trained model based on VIPL (Niu et al., 2019) is from the original open-source code of Physformer method. Additionally, the SCAMPS (McDuff et al., 2022b) as the only simulation database is used for comparing the results on real human and our 3D human.

### 4.2 Evaluation metrics

By using the above image based iPGG methods to process the videos of 3D models and real people respectively, we use two metrics to compare the performance of these iPPG methods running on 3D models and real people.

#### 4.2.1 Root-mean-square error

For real person, the Root-Mean-Square Error (RMSE) is used to measure the difference of heart rates calculated by the iPPG methods and the ground truth PPG sensor data recorded synchronously in public data sets. For 3D models, the RMSE refers to the difference between the heart rates extracted by the iPPG methods and the input pulse rate of the simulated cardiac signal. We computed a heart rate every 10 sec.

#### 4.2.2 Percent error

We use the percent error to evaluate the accuracy of the results from each iPPG method when running on real world videos and 3D human videos. The formula for percent error is Eq. 6:

$$\%Error=|\frac{P-T}{T}|\cdot 100\%$$ (6)

where *T* denotes to the ground truth heart rates in the tested public data set and the input heart rates of the simulated cardiac signal; *P* is the extracted values by the traditional methods and deep learning methods.

### 4.3 Results

In this part, the image-based iPPG methods described above were used to extract cardiac signals from both videos captured from 3D models and with real people in different scenarios. In order to verify the effectiveness and practicality of our method, we first compared the iPPG signal waveform recovered by the image-based methods then we compare the evaluation metric.

#### 4.3.1 Waveform of recovered iPPG signal and its frequency domain

**FIGURE 5:**
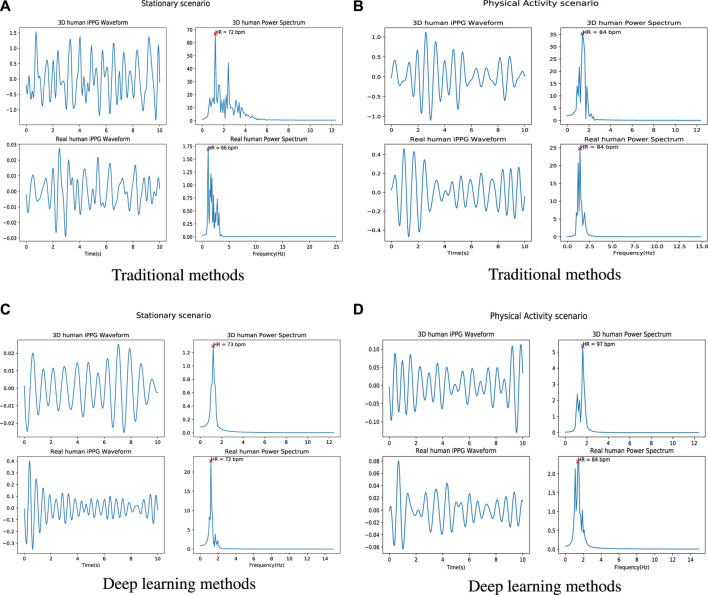
Examples of iPPG waveforms and their power spectra recovered from 3D/real human using traditional and deep learning methods. **(A)** is the iPPG waveform and its power spectra recovered by using traditional method (ICA) when 3D/real human under stable situation. **(B)** is the iPPG waveform and its power spectrum recovered using the traditional method (ICA) when 3D/real people perform physical activities. **(C)** is the iPPG waveform and its power spectra recovered by using deep learning method (Physformer) when 3D/real human under stable situation. **(D)** is the iPPG waveform and its power spectrum recovered using the traditional method (Physformer) when 3D/real people perform physical activities.

Figure 5 shows an example of the iPPG signal and its frequency domain extracted from videos of real people and 3D models under different scenarios (stable and active) by traditional method and deep learning method respectively. We found that the distribution of the 3D human pulse signal in the frequency domain looks qualitatively similar to that of a real person: 1) in the stationary scene (Figures 5A, C), compared with the iPPG signal processed by traditional methods, the pulse spectrum of the iPPG signal extracted by the deep learning method, both the pulse spectrum of the real person and the 3D person show less noise (especially can be observed in 5c); 2) for a person engaged in physical activity (Figures 5B, D), obvious regular movements can be observed in the low-frequency area (0.5–1 Hz) of the pulse spectrum of real people and 3D characters.

More comparison of raw signals from different scenarios can be found in Supplementary Figure S1. From the 3D human video, we can find fluctuations in the raw signal caused by expressions and lighting changes (see the purple dashed box in the 3D human scene), which is similar to the raw signal in the real world (see the yellow dashed box in the real human scene). Furthermore, since the entire cardiac cycle is a four phase activity, the fundamental frequency and its harmonic frequencies can be observed in the time-frequency diagrams of the real person (Supplementary Figures S2, S3). Based on this observation, from the time-frequency diagrams of the 3D human, we can see that the iPPG signal extracted from the 3D human can replicate the real signal.

#### 4.3.2 Comparison of the performances

**FIGURE 6:**
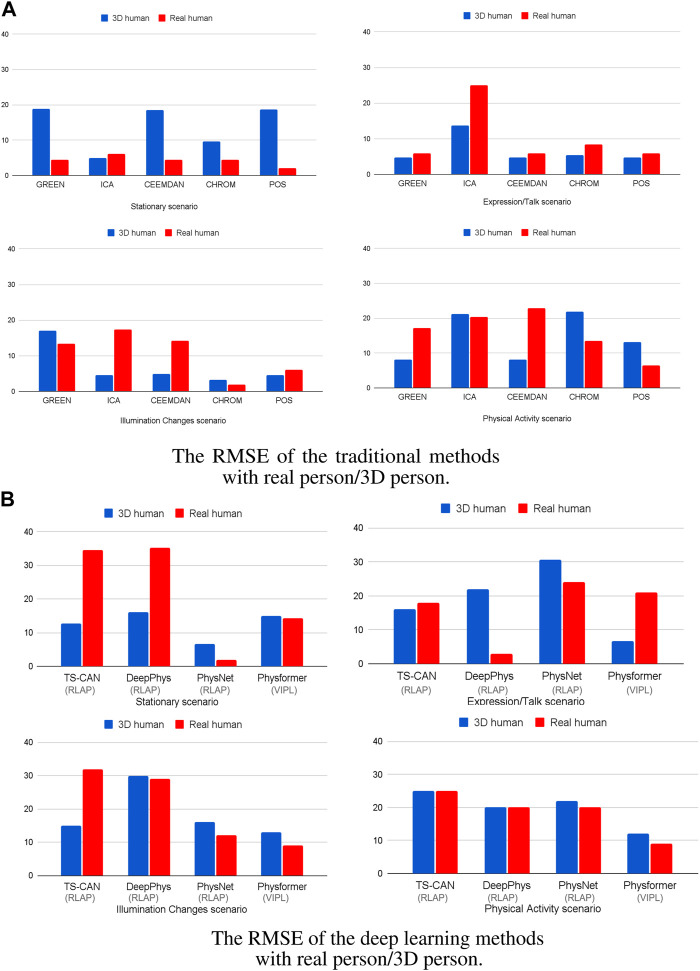
The RMSE of the **(A)** five traditional iPPG methods and **(B)** four deep learning methods with 3D/real human under four different scenarios. The gray brackets below the *x*-axis are the training sets of the deep learning model.

Figure 6 illustrates the RMSE for both real humans and 3D Human Models across four distinct scenarios. The data is processed by using the traditional methods (Figure 6A) and deep learning methods (on training set RLAP and VIPL) (Figure 6B), respectively. Body motions and darker skin types are both important factors effecting the accuracy of the recovered cardiac signals in many iPPG measurement algorithms. In Figure 6A, all the traditional iPPG methods show higher RMSE when analyzing real humans and 3D humans in motion, consistent with the fact that body motions are a problematic source of noise in image-based vital sign measurements. While in the stationary scenario in Figure 6A, except for ICA, the traditional methods do not perform well on 3D human videos. The high RMSE values are caused by dark skin tones, and if we remove the results for dark skin persons, all traditional methods show reliable performance in stable environments (RMSE all under 5 beats/min, see Supplementary Figure S4). We also notice that in the expression/talking scenario in Figure 6A, the ICA method shows the higher RMSE values both on real human videos (25 beats/min) and 3D human videos (14 beats/min) than other traditional iPPG methods, which is in line with the findings that ICA is more sensitive under the non-stationary scenario in De Haan and Jeanne (2013), Wang et al. (2016) and Al-Naji and Chahl (2018). For the performance of deep learning methods, in Figure 6B, it is obvious that the PhysNet algorithm outperforms the other methods both on real humans and 3D humans, especially in stationary scenes. This finding is in line with the results from Yu et al. (2019) who reported that a 3D-CNN version was able to achieve superior heart rate prediction errors comparing with a 2D-CNN architecture. Another interesting finding is that the performance of the Physformer method on both real humans and 3D humans are relatively stable in four different scenarios. It can be seen that both the traditional and deep learning iPPG methods have similar performance on real and 3D humans, which indicates that our method can maintain good agreement with real people under real-world conditions.

**FIGURE 7:**
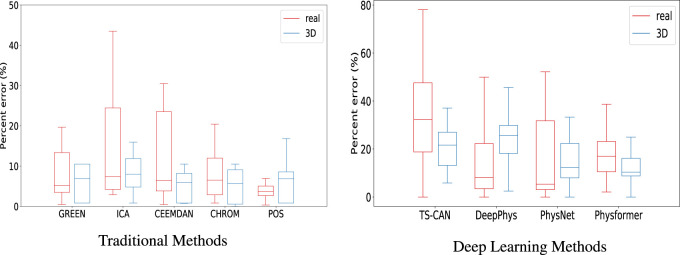
Boxplots showing the minimum, maximum, 25th-75th percentile, and median of the percentage error for **(A)** the five traditional iPPG methods and **(B)** the four deep learning methods run on real and 3D-rendered videos. The median values are indicated by red/blue bars inside the boxes, the quartile range by boxes, the full range by whiskers.

To further evaluate the agreement between the data sources, we then calculated the percent error (PE) for each method, the Boxplots of PE are shown in Figure 7. From the box plots of the percent error of the traditional methods, in Figure 7A, we can see that the median values of the two sets of boxes (real and 3D) are close on each method, especially on ICA, CEEMDAN and CHROM algorithm, which means that the average level of accuracy of the five traditional iPPG methods is similar when running on real human videos and 3D human videos. The large range of percentage error for the ICA method is caused by the high value of RMSE in Figure 6A, which we explained above. In particular, we can find that the iPPG method is more stable on 3D human videos, displayed as narrower boxes in both Figures 7A, B. This is also in line with the common understanding that the complexity of various factors in real-world scenarios which could affect the cardiac signal extraction is higher than that in simulated data.

Tables 3, 4 show the performance evaluation metrics (RMSE and PE) of traditional and deep learning methods on real and 3D humans. Since the deep learning “trained” model is usually not generalizable, from the evaluation metrics tables, we can see that traditional iPPG methods show stronger stable performance on both real and 3D humans. In particular, the deep learning models training on synthetic data set, SCAMPS does not perform well either on real humans or 3D humans. This result is also consistent across dataset test results of SCAMPS in (McDuff and Blackford, 2019; McDuff et al., 2022a; Wang et al., 2023). Although McDuff et al. (2022a) has reported that the models trained on combined real and synthetic data can improve heart rate extraction accuracy, how to make simulated data accurately supplement the lacking variables in different real-person datasets still requires further exploration.

**TABLE 3:** Evaluation metrics real human.

Method	Training set	Test set—real human							
		Stable		Expression/Talk		Light changes		Physical activity	
		RMSE	PE (%)	RMSE	PE (%)	RMSE	PE (%)	RMSE	PE (%)
GREEN	-	4.36	4.48	5.92	2.81	13.41	18.68	17.25	7.35
ICA	-	6.08	4.48	25.09	29.44	17.37	22.30	20.34	1.08
CEEMDAN	-	4.36	4.48	5.92	2.81	14.24	20.41	22.88	9.32
CHROM	-	4.38	6.55	8.38	9.00	1.94	2.73	13.49	13.62
POS	-	2.13	3.70	5.92	2.81	5.99	6.97	6.45	1.08
TS-CAN	RLAP	34.54	47.63	18.34	26.05	32.31	51.87	24.92	19.27
	SCAMPS	36.51	52.27	31.56	36.91	52.34	84.94	11.35	8.96
DeepPhys	RLAP	18.09	21.73	2.87	30.97	29.67	31.39	20.81	18.24
	SCAMPS	35.21	47.01	29.73	40.20	16.62	19.99	15.78	15.42
PhysNet	RLAP	2.06	2.56	24.82	29.82	12.18	12.18	19.51	15.16
	SCAMPS	27.95	36.39	28.03	31.75	19.39	29.55	24.85	25.21
Physformer	VIPL	14.31	18.46	20.62	25.89	9.46	14.54	9.20	7.95

**TABLE 4:** Evaluation metrics 3D human.

Method	Training set	Test set—3D human							
		Stable		Expression/Talk		Light changes		Physical activity	
		RMSE	PE (%)	RMSE	PE (%)	RMSE	PE (%)	RMSE	PE (%)
GREEN	-	18.85	5.31	4.76	4.25	16.99	20.09	8.18	6.74
ICA	-	4.93	5.92	13.69	17.08	4.5	5.60	21.14	14.63
CEEMDAN	-	18.55	2.83	4.76	4.25	4.96	6.16	8.18	6.74
CHROM	-	9.67	3.42	5.33	3.91	3.30	3.19	21.79	16.74
POS	-	18.74	5.31	4.76	4.25	4.54	6.17	13.07	10.95
TS-CAN	RLAP	12.77	17.87	28.39	32.92	14.69	17.47	25.33	24.76
	SCAMPS	25.01	24.50	20.76	21.62	24.24	34.39	32.28	23.01
DeepPhys	RLAP	16.12	24.08	22.02	27.66	30.59	43.34	20.48	18.37
	SCAMPS	25.01	24.50	23.97	25.79	41.13	53.83	32.28	23.01
PhysNet	RLAP	6.73	18.86	4.16	5.85	15.87	20.80	23.19	21.73
	SCAMPS	24.45	28.99	8.38	9.11	25.45	37.42	22.67	18.49
Physformer	VIPL	15.01	16.04	6.75	7.14	12.96	18.03	11.91	9.57

#### 4.3.3 Performance on fine-tuning model

**FIGURE 8:**
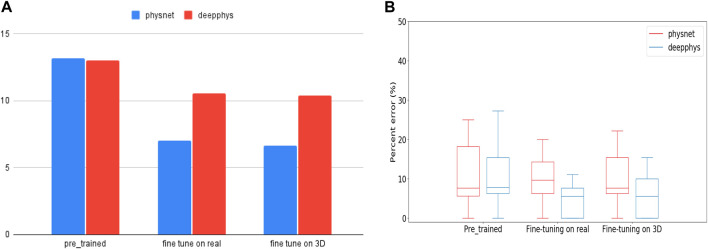
**(A)** is RMSE values represent the performance of pre-trained and fine-tuned models when tested on human doing physical activity. **(B)** is the boxplot of PE (Percent Error) for pre-trained and fine-tuned models during testing on human doing physical activity.

To investigate this work, we first used PhysNet and DeepPhys pre-trained models (trained on the RLAP dataset) to measure hear rates from real people performing physical activities (the test are data from MMPD dataset). Given that the RLAP dataset lacks physical activity features and contains subjects with similar skin tones, we fine-tuned the pre-trained model on real and synthetic dataset respectively. Subsequently, we evaluate the fine-tuned model on the same group of data on which we tested the pre-trained models. The real data for fine-tuning the models are walking people with diverse skin tones from MMPD dataset. The synthetic data for fine-tuning the models are our walking or running 3D humans with different skin tones. From Figure 8A, the Root Mean Square Error (RMSE) in heart rate estimates for models fine-tuned on real people is 7.05 bpm (Physnet) and 10.56 bpm (DeepPhys). Meanwhile, for models fine-tuned on 3D human data, the RMSE values are 6.66 bpm (Physnet) and 10.38 bpm (DeepPhys). Both scenarios demonstrate a performance improvement compared to the RMSE in heart rate estimates on the pre-trained model, which is 13.16 bpm (Physnet) and 12.99 bpm (DeepPhys). Also from the Figure 8B we can see that the fine-tuned model exhibits a reduction in error and outperforms in terms of stability. These findings indicate that our 3D human can help to enhance the generalization capabilities of deep learning models.

## 5 Discussion

Our results show it is possible to build data sets based on 3D Human Models with simulated cardiac signals and involuntary body movements for testing image-based iPPG method or to supplement the training set. The raw signals and cardiac signals of videos with 3D humans were quite similar to the signals from videos with real humans. The distributions in the frequency domain of the 3D human’s cardiac signals are qualitatively similar to the distributions found with real humans. The comparison of evaluation metrics (RMSE and PE) show that the tested traditional and deep learning iPPG methods have similar performance while running on real world videos and the simulated videos using our model. These experiments are intended to demonstrate that rendered videos of 3D models closely match videos of real people. In addition, this also shows that since all variables in the model can be imported into video rendering using python, it is possible to precisely control the input variables. Rendering our 3D human in different environments can be used for iPPG algorithm validation and noise analysis in future research. Futhermore, since our 3D human has a complete body and skeleton (rig), any pose can be created and rendered in any scene. This function can be applied to some areas that are difficult to achieve with real-person data sets, such as human vital sign detection based on iPPG method in drone rescue.

## 6 Conclusion

We have undertaken a novel study that simulates the cardiac signal on a 3D Human Model. In order to enhance the authenticity of the entire model and the possibility of adding more variables in the future, we also added environmental variables such as lighting changes and body movement to the rendered video. Five well-known traditional iPPG method and four deep learning iPPG method have been used to process the rendered videos and the results were compared with those extracted from real human videos. The results section shows that the signals from the 3D human in both the time domain and frequency domain have good agreement with the data from the comparison group (videos with real humans). The future direction of this study will be adding more environmental conditions and integrating more advanced vital signs such as HRV analysis, SpO2 and blood pressure into 3D Human Models. More exploration in some specialised fields, such as simulated drone rescue scenes and synthetic patients’ physiological signals will be conducted in the future.

## Data Availability

The original contributions presented in the study are included in the article/Supplementary Material, further inquiries can be directed to the corresponding author.
