# Supplementary material for: Simulating cardiac signals on 3D human models for photoplethysmography development
Source: Front Robot AI. 2024 Jan 10;10:1266535. doi: 10.3389/frobt.2023.1266535 (PMC10806157; doi:10.3389/frobt.2023.1266535)
Supplement: Supplementary file 1 [file DataSheet1.PDF]

frontiers

Supplementary Material

1 SUPPLEMENTARY DATA

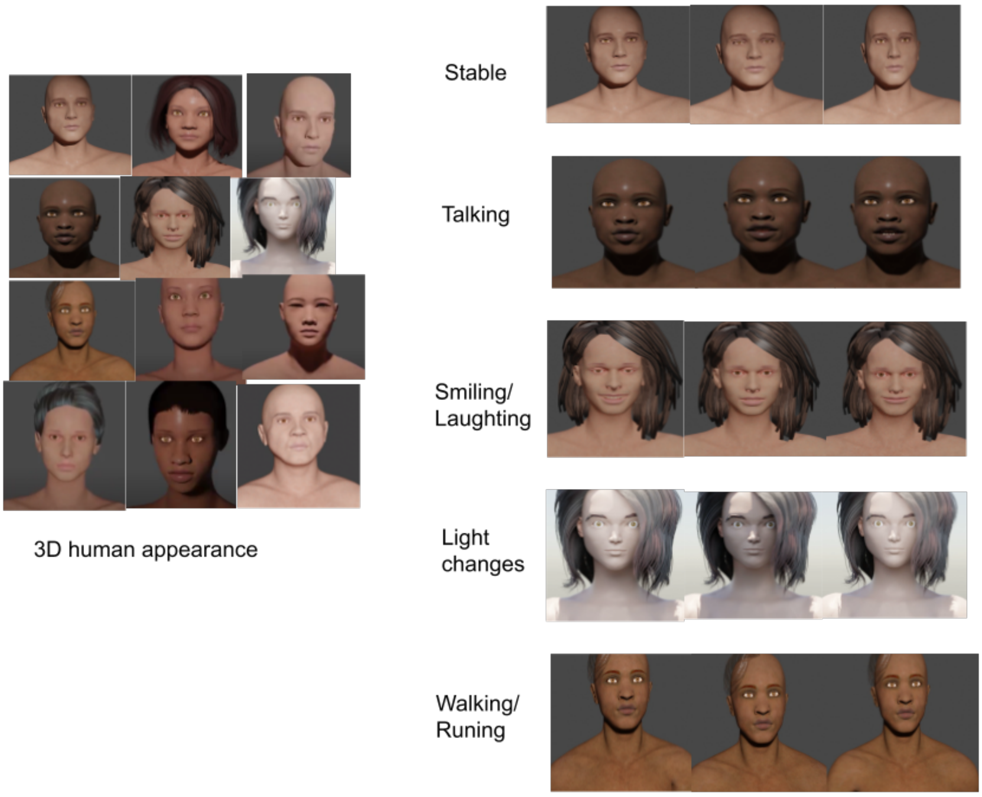

Figure S0.

The 3D human videos dowload link: data

Lable/number	L/3<br>light change	S1/3<br>stable in lab	S2/2<br>stable in nature light	
	F1/3<br>facial action in lab	F2/3<br>facial action in nature	E1/10<br>body motion in lab	E2/10<br>body motion in nature

Table S1. Distribution
